# Cytokine-induced memory-like natural killer cells for cancer immunotherapy

**DOI:** 10.1186/s13287-021-02655-5

**Published:** 2021-12-04

**Authors:** Mubin Tarannum, Rizwan Romee

**Affiliations:** Division of Cellular Therapy and Stem Cell Transplantation, Dana Farber Cancer Institute, Harvard Medical School, 450 Brookline Ave, Boston, MA 02215 USA

**Keywords:** Natural killer cells, Cytokine-induced memory-like NK cells, Innate memory, Cancer immunotherapy, Adoptive cell therapy

## Abstract

Natural killer cells are an important part of the innate immune system mediating robust responses to virus-infected and malignant cells without needing prior antigen priming. NK cells have always been thought to be short-lived and with no antigen specificity; however, recent data support the presence of NK cell memory including in the hapten-specific contact hypersensitivity model and in certain viral infections. The memory-like features can also be generated by short-term activation of both murine and human NK cells with cytokine combination of IL-12, IL-15 and IL-18, imparting increased longevity and enhanced anticancer functionality. Preclinical studies and very early clinical trials demonstrate safety and very promising clinical activity of these ***c***ytokine-***i***nduced memory-like (CIML) NK cells, making them an attractive cell type for developing novel adoptive cellular immunotherapy strategies. Furthermore, efforts are on to arm them with novel gene constructs for enhanced tumor targeting and function.

## Introduction

Natural killer (NK) cells are a type of innate lymphocytes important for mediating anti-viral and anti-tumor responses [1, 2]. NK cells are unique as they exhibit rapid and potent cytolytic activity against virus-infected and malignant cells without requiring prior antigen exposure [1, 3]. Conventional NK cells constitute approximately 5–15% of the peripheral blood lymphocytes and have a short half-life compared to the B and T lymphocytes and therefore need constant replenishment. In the peripheral blood, approximately 90% of the NK cells are mature CD56^dim^CD16^+^, while approximately 10% are immature CD56^bright^CD16^±^ [4, 5].

**Fig. 1:**
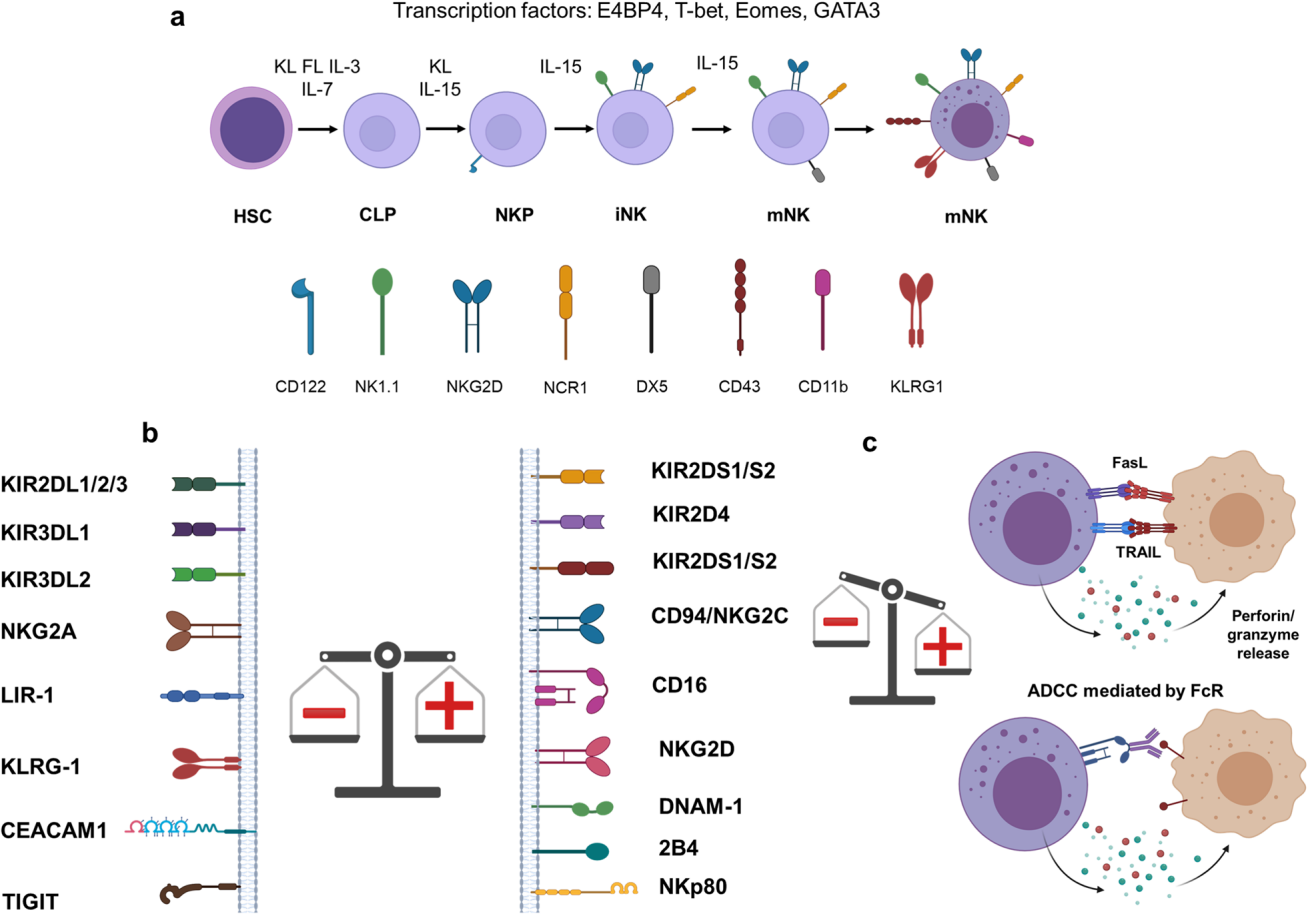
Schematic representation of the NK cell development, activation and cytotoxicity. **a** Developmental stages of NK cells, hematopoietic stem cells (HSC), common lymphoid progenitor (CLP), natural killer progenitor (NKP), immature NK (iNK) and mature NK (mNK) cells. **b** The NK cell function is controlled by the expression of inhibiting (left) and activating receptors (right). **c** Once the net balance is shifted toward the activating signals, NK cells kill their targets through the release of perforin/granzyme, FasL- and TRAIL-mediated apoptosis and antigen-dependent cellular cytotoxicity (ADCC)

NK cells develop from CD34^+^ hematopoietic progenitor cells in the bone marrow, and their differentiation is thought to be completed in the peripheral lymphoid tissues (Fig. 1) [6–8]. Differentiation of NK cells from the hematopoietic stem cells progresses through multiple stages and arbitrarily divided into five stages, including hematopoietic stem cells (HSCs), common lymphoid progenitors (CLPs), natural killer progenitor cells (NKPs), immature NK cells (iNKs) and mature NK cells (mNKs). This process requires a combined effect of multiple transcription factors and cytokines (Fig. 1a) [9–12]. In contrast to the B or T cells, NK cells do not rearrange genes to acquire clonally arranged antigen-specific receptors. NK cell function is dictated by a delicate balance of activating and inhibitory signals from an array of germline DNA-encoded activating and inhibitory cell receptors [4, 5]. NK cells must thus express at least one inhibitory receptor specific for self-MHC class I to attain licensing (arming/education) and to ensure tolerance [13, 14]. Among these inhibitory receptors, killer cell Ig-like receptors (KIRs) and NKG2A recognize MHC I and MHC I-like molecules on the healthy cells and thus prevent auto-reactivity/maintain tolerance [15]. Key activating receptors expressed by the NK cells include CD16, NKG2D, NKp46 and 2B4 [13] (Fig. 1b). CD16 (FcγRIIIa) binds to the Fc part of the IgG molecules mediating antibody-dependent cellular cytotoxicity (ADCC) [16, 17]. Once threshold for net activation is reached, an NK cell can kill the target cell through multiple mechanisms. CD56^dim^ NK cells have a higher cytotoxic function against tumor targets at baseline, whereas the CD56^bright^ NK cells are responsible for late innate inflammatory activity through IFNγ, TNFα, G-CSF, GM-CSF and IL-3 [18]. NK cells utilize two main mechanisms for cell cytotoxicity, granule exocytosis and death receptors. For granule exocytosis, cytotoxic granules containing perforin and granzyme are released into the immune synapse [19]. By forming pores in the cell membrane, perforin facilitates granzyme entry into the target cells where these proteases cleave targets to induce apoptotic cell death. Receptors including Fas ligand (FasL) and tumor necrosis factor-related apoptosis-inducing ligand (TRAIL) bind to their ligands on target cells and thus induce apoptosis (Fig. 1c) [20]. NK cells also participate in a complex network of interactions with other key immune cells including dendritic cells (DCs), T and B cells to prime adaptive immunity through cytokines and chemokines [21].

In recent years, immunotherapy has ushered a major monumental shift in the paradigm of cancer therapy. Therapeutic potential of NK cells was initially recognized based on their ability to enhance graft versus leukemia (GvL) effect while avoiding graft versus host disease (GvHD) in the setting of HLA-haploidentical hematopoietic cell transplantation (HCT) [22]. NK cells are negatively regulated by major MHC I-specific inhibitory receptors, where in a given individual specific KIR^+^ NK cells are blocked by their cognate class I alleles. Missing expression of the KIR ligands on the mismatched allogenic tumor cells triggers NK cell alloreactivity. The fine tuning of the NK cell function by activating receptors binding cognate ligands on the tumor cells and lack of inhibition by inhibitory receptors allows NK cells to recognize and target allogeneic tumor cells [23], and this contributes significantly to the GvL effect in HLA-haploidentical HCT setting [24].

NK cells are the first cell type to recover after HCT and therefore may modulate GvHD in addition to their effect on the GvL effect. NK cells may help prevent GvHD by repressing alloreactive APCs, as well as by direct killing of the alloreactive T cells [25, 26]. The fined-tuned inhibitory and activation receptors on the NK cells may help prevent NK cells from causing GvHD as activation signals are required along with the loss of inhibition to cross the activation threshold of these cells. Theoretically, production of pro-inflammatory cytokines by NK cells could, however, promote direct tissue damage and or indirectly increase T-cell-mediated tissue damage in post-transplant setting [27]. However, there is no substantial evidence which supports role of NK cells in mediating GvHD after allogeneic HCT.

These observations have significantly helped generate interest in using adoptive NK cell-based immunotherapy approaches in advanced cancer [28]. Advantages of NK cells over other immune cells for cancer immunotherapy include quick and potent response, lack of GvHD, minimal to no cytokine release syndrome (CRS) and no neurotoxicity [29, 30]. Importantly, NK cells can kill 3–4 tumor cells and with a subpopulation capable of serial killing (may kill 30 or more tumor cells) [8, 31]. However, poor persistence, expansion and exhaustion of the adoptively transferred NK cells remain some of the major challenges in the field [20, 28].

## Innate memory in NK cells

Immunological memory is a critical evolutionary feature defined as the ability to remember previous antigen encounter and mediate qualitatively and quantitatively increased responses upon secondary exposure [32]. This immune cell memory is traditionally associated with conventional adaptive immune system, including B and T lymphocytes. Immune memory involves antigen-dependent clonal proliferation and their capacity to persist for long time, sometimes lifetime. In the B and T cells, this is achieved by gene rearrangement, somatic hypermutation process (SHM), isotype switching and class switch recombination (CSR) increasing antigen receptor affinity [33, 34]. Recent studies have supported the presence of key immune memory features in innate immune cells referred to as adaptive or trained immunity [35]. Effector cells of innate immune system, including monocytes and macrophages, have been shown to mediate this trained immunity with long-lasting altered responses to the second stimulation. Trained or adaptive immunity in innate immune system can be non-specific or antigen specific [35, 36].

NK cells have long been considered short-lived, non-specific, and not being able to remember prior antigen/cytokine exposure. Recently, numerous paradigm changing studies have supported the presence of memory and memory-like functions in NK cells [37–41]. The NK cell memory has been studied in three main scenarios: hapten specific, virus specific and cytokine induced [42, 43]. NK cell-mediated antigen-specific memory was first observed in a murine model of hapten-mediated contact hypersensitivity (CHS) [38]. Rechallenge in hapten-sensitized Rag2 (recombination activating gene 2)-deficient mice lacking both T and B cells induced CHS responses and thus also demonstrate that NK cells are both required and sufficient to mediate this response. The CHS response observed was antigen-specific, persisted for a long time, and importantly, the memory features could be transferred by adoptive transfer of hepatic NK cells to naïve mice [38]. NK memory has also been demonstrated in response to viruses in mice and humans [43].

Early studies involving murine cytomegalovirus (MCMV) infection showed that murine NK cells acquire traits of adaptive immunity post-infection [39]. The m157 glycoprotein on MCMV-infected cells is recognized by the Ly49H receptor expressed on a murine NK cell subset and selective expansion of Ly49H^+^ NK cell subset was observed [39, 44]. Similar to MCMV, human cytomegalovirus (HCMV) is also able to induce expansion of an NK subpopulation expressing NKG2C receptor where the antigen on the surface of HCMV is not known yet [45, 46]. CD56^dim^CD16^high^NKG2C^+^ cells exhibited enhanced proliferative capacity and cytokine secretion post-HCMV exposure. Increased expansion of CD56^dim^CD16^high^NKG2C^+^ NK cells has also been found in patients with other viral infections including hepatitis C, HBV, EBV and HIV-1, however, only in patients with prior HCMV infection [47]. HCMV-specific NK expanding subset not only expresses NKG2C, but also HLA-I-specific KIRs and CD57 [48, 49]. Furthermore, they also have decreased the expression of FcɛRγ, SYK and EAT-2 proteins [46]. At the molecular level, NK cell memory to CMV has been correlated with modified chromatin states, modulated DNA methylation levels which are reduced at the IFNγ gene locus and more accessible chromatin at the effector genes [50, 51]. Independently, studies in influenza-induced long-lived NK cells led authors to conclude that cytokine activation alone is likely sufficient in generating long-lived memory features [52].

In addition to the direct involvement of cytokines in the differentiation and function of NK cells, inflammatory cytokines can also endow memory-like features in murine and human NK cells in the absence of an antigen, termed as cytokine-induced memory-like (CIML) NK cells [40, 41, 53]. Though there have been reports of memory-like functionality after CD16A engagement [54], in the current review we will focus primarily on the CIML NK cells. The CIML NK cells are antigen non-specific; they possess enhanced proliferative capacity and exhibit prolonged persistence in vivo. Cooper et al. described murine CIML NK cells demonstrating NK cells stimulated briefly with a specific cytokine combination of IL-12, IL-15 and IL-18 and resulted in persistence and enhanced IFNγ even after 4 months of adoptive transfer into Rag1−/− mice [40, 55]. These cells also exhibited increased IFNγ production upon re-stimulation ex vivo associated with stable intrinsic demethylation of conserved noncoding sequence (CNS1) in the IFNγ locus.

The NK memory features were also successfully demonstrated in human NK cells after a brief (12–18 h) preactivation with the IL-12, IL-15 and IL-18 cytokine cocktail [41]. Brief preactivation, followed by a prolonged rest period in vitro, resulted in enhanced IFNγ production upon restimulation with K562 leukemia cells evident in both CD56^bright^ and CD56^dim^ NK cell subsets. In addition, detailed immunophenotyping revealed increased expression of CD94 and NKG2A in CD56^dim^ NK cells, and NKp46 and CD69 in CD56^bright^ and CD56^dim^ subsets. NK cell surface marker analysis showed a positive correlation between IFNγ production and expression of CD94-, NKG2A-, NKG2C- and CD69-preactivated CD56^dim^ NK cells [41]. In relation to GvHD, adoptive transfer of murine CIML NK cells showed suppression of GvHD, while GvL effect was maintained in fully mismatched murine HCT setting [56, 57]. The same study detailed the loss of Eomes and T-bet after adoptive transfer in IL-2-activated NK cells (control), whereas CIML NK cells maintained the expression in the GVHD model. The Eomes and T-bet expression was linked to the prolonged proliferation and cytolytic potential of CIML NK cells as well as to their anti-GvHD role in this model [56, 57]. Similarly in the clinical setting, the first-in-human clinical trial did not result in GvHD as described below, though exact mechanism(s) needs to be further investigated [58]. Furthermore, both self- (licensed) and non-self (un-licensed) CIML NK cells have similar anti-tumor responses, suggesting that the traditional KIR/KIR-ligand mismatch may not fully apply to these cells [59]. Also, engagement of CD16 on non-self (unlicensed) CIML NK cells leads to the enhanced anti-leukemia responses through ADCC mechanism [59].

Recently, Smith et al. demonstrated seven subsets of NK cells including a small fraction with hybrid features of CD56^dim^ and CD56^bright^ in the peripheral blood from healthy volunteers using single-cell RNA sequencing [60]. Most prominently, these cells showed significant upregulation of granzyme after IL-2 treatment, an important feature of enhanced recall response in CIML NK cells [60]. Apart from the viral infections, NK cells also acquire cell-intrinsic memory-like properties, after endotoxemia [61]. The memory NK cells were maintained for up to 9 weeks even in a suppressive immune environment showing the persistence and advantages of memory NK cells.

**Fig. 2:**
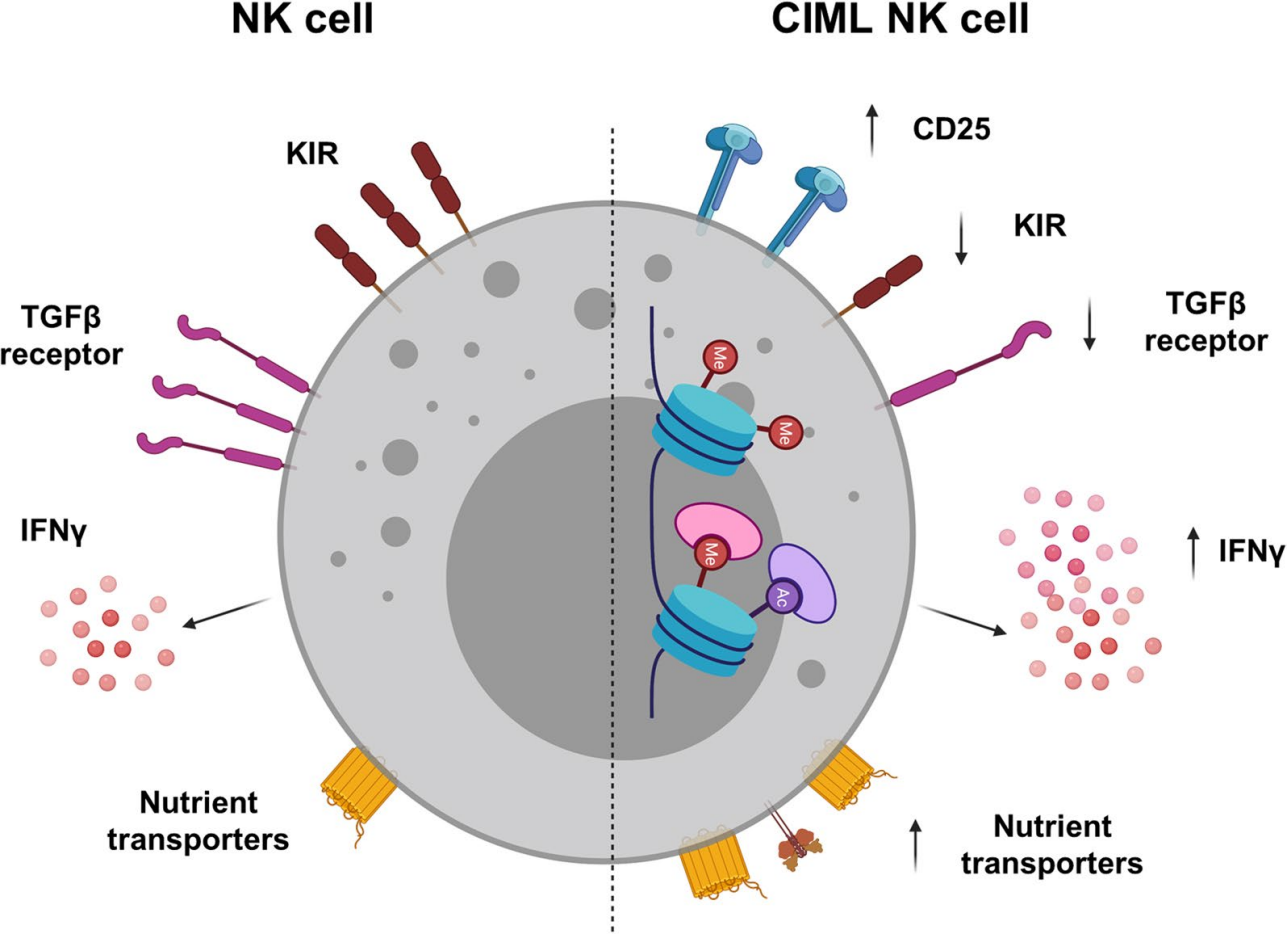
Schematic representation of the features observed in cytokine-induced memory-like (CIML) NK cells. Key CIML NK cell features include increased expression of CD25 (IL-2Ra), decreased expression of KIRs, and TGFβ receptors which may help unleash the inhibition in CIML NK cells. Increased IFNγ production may contribute to the enhanced anti-tumor responses. Epigenetic changes like CpG demethylation have been reported, and others need further investigation. Metabolic changes in CIML NK cells including glucose transporter and transferrin receptor play an important role in the long-term persistence and recall functions

The mechanisms involved in the CIML differentiation and contributing to their key properties are not fully understood currently. It is known that cytokines like IL-12, IL-15 and IL-18 downregulate the TGF-β receptor and its signaling pathway which may contribute to the enhanced anti-tumor responses by the CIML NK cells (Fig. 2) [62]. Additionally, study by Ewen et al. revealed that stimulation of NK cells with IL-12/15/18 results in downregulation of KIR (KIR2DL2/L3, KIR2DL1 and KIR3DL1) which reduces their sensitivity to self-HLA-I inhibition [63]. Ghofrani et al. also showed that semaphorin 7A (SEMA7A) is upregulated on CIML NK cells which correlated with IFNγ production. This study illustrated a novel mechanism of SEMA7A/integrin β1 interaction playing an important role in the CIML NK cell differentiation [64]. Various epigenetic changes may also contribute to the long-term persistence of CIML NK cells that overlap with observations made in virus-specific NK cell memory: demethylation of the IFNγ locus, CpG demethylation of the PRDM1/BLIMP1 and ZBTB32/TZFP genes or hypermethylation of the FCER1G gene as detected in the NKG2C^+^ adaptive NK cells, which needs to be studied in detail in the CIML setting [65]. Consider stable epigenetic changes are found in the adaptive NKG2C + NK cells, which are similar to those observed in the memory T cells [50]. This includes transition states from naïve to memory development and common epigenetic programs in adaptive memory-like NK and CD8 memory T cells, including Bach2, Tcf7, and Zeb2, tox and Themis2. Therefore, CIML differentiation may include alterations similar to those observed in the memory T cells; however, these need to be evaluated in future studies [50, 66].

Cytokine activation of NK cells can also lead to elevated oxidative phosphorylation (OXPHOS) and elevated glycolysis [44]. In fact, increased glycolysis was shown to be correlated with MCMV-mediated adaptive NK cells [67], pointing to the metabolic changes in the NK cells that may play a vital role in their effector functions and recall responses [44]. A very recent study by Terrén et al. showed that expression of nutrient transporters including transferrin receptor (CD71), amino acid transporter (CD98) and glucose transporters (GLUT1 and GLUT3) was increased after NK cells were exposed to CIML conditions [68]. Importantly, the CIML NK cells preserved elevated expression of amino acid transporters even in the resting phase. The study also reported that CIML NK cells undergo metabolic switch toward glycolysis which persists even after the cytokine withdrawal. Detailed analysis of the relation between glycolysis and effector function showed that the production of IFNγ and MIP-1β was sensitive to glycolysis inhibition, whereas other effector functions including TNFα secretion, degranulation and cytotoxic activity were not very sensitive to the glycolysis inhibition [68].

## Harnessing NK cell memory for enhanced immunotherapy

CIML NK cells are attractive for adoptive cell therapeutic approaches due to their key characteristics which include anti-tumor responses as well as prolonged proliferation and persistence in vivo [69]. In a preclinical mouse model by Ni et al., adoptive transfer of IL-12/15/18-preactivated murine NK cells combined with irradiation substantially reduced the growth of established mouse tumors [53]. NK cell infusion in MHC class I-deficient RMA-S cell-bearing tumor models combined with sublethal dose of radiation (5 Gy) significantly reduced the tumor growth and prolonged survival of mice. Increased therapeutic benefit of preactivated NK cells was also demonstrated in a metastatic lung model of melanoma in this study. Infiltration of the infused immune cells into the tumor microenvironment is critical for successful immunotherapy, and CIML NK cells exhibited strikingly higher numbers in the tumors compared with IL-15-pretreated NK cells [53]. Similarly, human CIML NK cells maintained memory-like phenotype in a preclinical xenograft model and also demonstrated improved control of human leukemia cells in vivo [58]. Uppendahl et al. evaluated CIML NK cells against ovarian cancer, and they showed enhanced cytokine production and killing of the ovarian cancer cells by CIML NK cells compared to the conventional NK cells [70]. In this study, CIML NK cells also exhibited enhanced effector functions in the immunosuppressive microenvironment with potent antitumor responses.

Based on the promising in vitro and in vivo activity of CIML NK cells, our group did a first-in-human phase 1 clinical trial of CIML NK cells in patients with relapsed/refractory acute myeloid leukemia (AML) (clinicaltrials.gov # NCT01898793). In this study, CIML NK cells were generated from conventional NK cells from related haploidentical donors using 12- to 16-h activation with IL-12, IL-15 and IL-18 before their adoptive transfer. In this trial, a 2-step CD3 depletion followed by CD56-positive selection using Miltenyi CliniMACS was used for enriching NK cells consistently yielding > 90% NK cell purity. CIML NK cells were infused after the patients received several doses of fludarabine and cyclophosphamide for lymphodepletion (while some previous studies have also used total body irradiation for enhanced NK cell expansion [71]). The patients also received low-dose IL-2 (1 × 10^6^ IU/m^2^ every other day × 7) subcutaneously which was very well tolerated. A total of 19 patients were treated on this study, and none of the evaluable patients had any major toxicity including cytokine release syndrome (CRS) or GvHD. We saw very promising results with > 50% of the patients being able to clear the blasts despite most of the patients having high blast content in their bone marrow and without manifesting any major toxicity. Despite using allogeneic (haploidentical) CIML NK cells in this study, we observed robust proliferation, expansion and sustained enhanced anti-leukemia activity after adoptive transfer of these cells [58]. Adoptively transferred CIML NK cells exhibited phenotypically distinct phenotypical markers compared to the baseline NK cells with significantly increased activating receptors NKG2D, NKp30 and NKp44 as well as CD69, CD62L and NKG2A [72]. Interestingly, CIML NK cells post-adoptive transfer did not express CD25/IL-2Rα in contrast to the CIML NK cells expanded in vitro. The analysis also demonstrated that NKG2A was transcriptionally upregulated on CIML NK cells and associated with treatment failure in these patients [72]. Though this study included infusion of IL-2 due to the higher affinity of CIML NK cells to IL-2 [73], future approaches may involve replacing IL-2 with IL-15. IL-15 is a cytokine important for differentiation, homeostasis and activation of the NK cells and also importantly does not expand regulatory T cells [74]. An IL-15 mutein bound to the IL15Ra sushi domain attached to the IgG1 (N-803) has also been used in recent studies to activate and expand NK cells in vivo and is an attractive molecule for combining with CIML NK cell-based adoptive transfer studies in future [75–78].

Based on the promising activity as well as safety of using CIML NK cells in the above-mentioned study, there is a significant interest in the field to expand their use to other tumor settings. We are currently evaluating the use of CIML NK cells in patients with myeloid malignancies relapsed after haploidentical hematopoietic stem cell transplantation (Clinicaltrials.gov # NCT04024761). In this study, CIML NK cells are generated from the original stem cell donor and thus making CIML NK cells potentially immune compatible with the circulating T cells. The latter should favor in vivo expansion and persistence of the adoptively transferred CIML NK cells. Relapse after hematopoietic stem cell transplantation is an unmet need as the use of donor lymphocyte infusion (DLI) commonly used to treat these patients is associated with low response rates and putting the patients at a risk of developing GVHD. Furthermore, patients with acute leukemia with minimal residual disease (MRD) in the peri-transplant period have significantly higher risk of disease and therefore maybe best suited for NK cell-based interventions to prevent disease relapse in future studies [79].

We are also evaluating potential safety and potential efficacy of allogeneic CIML NK cells in patients with advanced metastatic head and neck cancer (Clinicaltrials.gov # NCT04290546). In this study, the patients are also given a dose of CTLA-4 blocker ipilimumab prior to the CIML NK cell adoptive transfer aimed at depleting intratumoral regulatory T cells [80]. Furthermore, to further enhance their in vivo activation, proliferation and persistence, the patients receive IL-15 super-agonist which preferentially activated NK (and conventional CD8 T cells) without significantly affecting regulatory T cells [78].

**Fig. 3:**
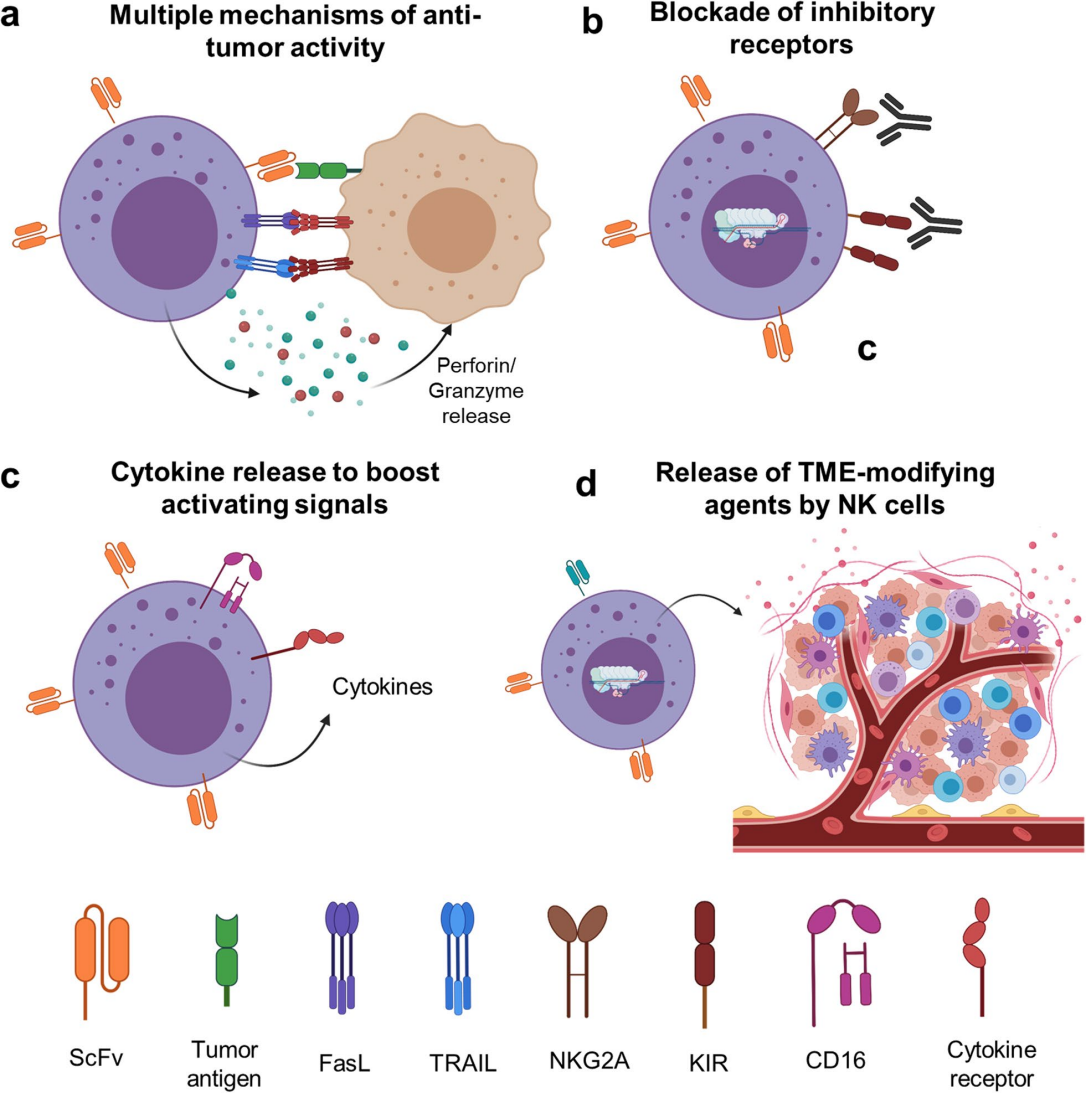
Overview of strategies to improve cancer therapies using CIML and CAR-CIML NK cells. **a** CIML NK cells can exhibit anticancer activity via multiple mechanisms including CAR activation, ADCC and stress-related signals on tumor cells. **b** The activity of CIML and CAR-CIML cells can be further enhanced by using antibody or CRISPR-based strategies to block checkpoint pathways like NKG2A. **c** The activating signals can be boosted by increasing their expression as well as CAR-CIML NK cells can be engineered to release cytokines and other activating molecules for increased persistence and cytotoxic activity. **d** CAR-CIML NK cells can also be engineered to release TME-modulating agents like IL-12 for promoting a pro-inflammatory milieu

CIML NK cells are a perfect platform for developing chimeric antigen receptor (CAR) NK cells based on their favorable safety profile, increased proliferation, prolonged persistence and enhanced as well as various modes of antitumor function seen in vivo in preclinical animal models and in patients treated with genetically un-modified CIML NK cells. Recent study by Gang et al. has demonstrated the feasibility and efficacy of CAR-CIML NK cells against NK-resistant B-lymphoma malignancy in vitro and in a xenograft mouse model [81]. CD19-CAR CIML NK cells with the second-generation CAR with 4-1BB and CD3ζ intracellular signaling domains demonstrated superior activity compared to the CD19-CAR control NK cells. The CAR-CIML NK cells showed increased IFNγ, degranulation and enhanced CD19-specific killing of the CD19^+^ Raji cells as well as primary patient-derived lymphoma cells. The CAR-CIML cells also expanded in vivo after adoptive transfer, resulted in a significant decrease in tumor burden and improved survival of treated mice [81]. Our group has recently developed CAR-CIML NK cells targeting a tumor-specific neoepitope expressed on the cell surface by HLA-A2 in NPM1-mutated AML [82]. Anti-NPM1 CAR significantly demonstrated anti-tumor function and specific killing against NPM1-mutated AML cell lines. We are currently generating preclinical data needed to support initiating a phase 1 trial of CAR-CIML NK cells in patients with relapsed/refractory NPM1-mutated AML. These studies demonstrate the feasibility and promise of using CIML NK cell-based approaches to further advance cancer immunotherapy. Other key approaches being pursued currently include CRISPR and non-CRISPR-based manipulations for decreasing inhibition by checkpoint pathways like NKG2A, increasing in vivo survival by incorporating novel CAR constructs capable of releasing cytokines for self-sustenance as well as releasing TME modulating cytokines like IL-12 (Fig. 3).

Development of NK cell and CIML NK cell therapies in solid tumors is currently a highly investigated field, even though the effectiveness of NK cell-based therapies in the solid tumor setting has been modest compared to the myeloid malignancies. This preferential propensity of NK cells to target myeloid malignancies is not fully understood, but may be related to the HLA class 1 and non-classical HLA class 1 molecules like HLA-E expression. In this context, creating a missing self-recognition via antibody-mediated disruption of pan-KIR2D or NKG2A/HLA-I interaction could potentially prove to increase the effectiveness of NK cells against solid tumors [83]; for example, the anti-NKG2A antibody, monalizumab, is in the clinical development currently (Fig. 3b) [84]. In addition, other strategies are also under investigation to increase the NK cell functionality in solid tumors [85]. Though there are different populations of immune cells including NK cells in the solid tumors, their functionality is extremely repressed due to factors like hypoxia, TGFβ and desmoplastic stroma [86]. Hence, additional strategies aimed at manipulating tumor microenvironment (TME) are required, for example, armoring CAR NK or CAR-CIML NK cells to express cytokines (like IL-12 and IL-15) and incorporation of TGFβ traps [87, 88]. Genetic manipulations to incorporate tumor microenvironment modulators (Fig. 3d) hold promise in adoptive cell therapies for solid tumors and were recently reviewed in detail by Chen et al. [89].

## Summary statement

The quick response of NK cells in combination with long-lasting memory features can be a huge asset to developing therapeutics in various diseases, importantly cancer. These memory-like features can be achieved by brief stimulation of cytokines. Recent proof toward the functionality of CIML NK cells in cancer is encouraging, and clinical data support the advantage of using CIML NK cells for immune therapy. Moving forward, the long-term persistent enhanced effector function of CIML NK cells can be combined with genetic engineering of CAR to increase their antigen specificity. This is already being explored with encouraging preliminary results.

## Data Availability

Not applicable.

## Abbreviations

NK cells: Natural killer cells
CIML NK cells: Cytokine-induced memory-like NK cells
HSCs: Hematopoietic stem cells
CLPs: Common lymphoid progenitors
NKPs: Natural killer progenitor cells
iNKs: Immature NK cells
mNKs: Mature NK cells
KIRs: Killer cell Ig-like receptors
ADCC: Antibody-dependent cellular cytotoxicity
TRAIL: Tumor necrosis factor-related apoptosis-inducing ligand
DCs: Dendritic cells
GvL: Graft versus leukemia
GvHD: Graft versus host disease
HCT: Hematopoietic cell transplantation
SHM: Somatic hypermutation process
CSR: Class switch recombination
CHS: Contact hypersensitivity
Rag2: Recombination activating gene 2
MCMV: Murine cytomegalovirus
HCMV: Human cytomegalovirus
CNS1: Conserved noncoding sequence
SEMA7A: Semaphorin 7A
OXPHOS: Oxidative phosphorylation
AML: Acute myeloid leukemia
CRS: Cytokine release syndrome
DLI: Donor lymphocyte infusion
MRD: Minimal residual disease
CAR: Chimeric antigen receptor
TME: Tumor microenvironment
